# Sustained-Release Corticosteroid Options

**DOI:** 10.1155/2014/164692

**Published:** 2014-07-23

**Authors:** Mariana Cabrera, Steven Yeh, Thomas A. Albini

**Affiliations:** ^1^Department of Ophthalmology, University of Miami Miller School of Medicine, Bascom Palmer Eye Institute, 900 NW 17th Street, Miami, FL 33136, USA; ^2^Department of Ophthalmology, Emory University, 1365 Clifton Road NE, Atlanta, GA 30322, USA

## Abstract

Sustained-release corticosteroid treatment has shown to be a promising strategy for macular edema due to retinovascular disease (i.e., diabetes and retinal vein occlusion) and for the treatment of noninfectious posterior uveitis. Clinicians now have the option of three sustained-release corticosteroid implants: Ozurdex (Allergan Inc., Irvine, CA) which releases dexamethasone and two devices that release fluocinolone acetonide, Retisert (Bausch & Lomb, Rochester, NY), and Iluvien (Alimera Science, Alpharetta, GA). Each has different physical characteristics and duration effect and has been approved for different indications. Herein we provide a summary of the current clinical knowledge regarding these implants.

## 1. Introduction

Intraocular corticosteroids are used for a variety of ophthalmologic conditions such as diabetic macular edema [1], posterior uveitis [2], and macular edema secondary to vascular occlusions [3]. Initially, triamcinolone acetonide was used [4], but many of these conditions are chronic and required repeat injections for prolonged periods of time. This is inconvenient to patients and may increase the risk of complications secondary to the injection procedure, including endophthalmitis and vitreous hemorrhage. Clinicians now have the option of three sustained-release corticosteroid implants: Ozurdex (Allergan Inc., Irvine, CA) which releases dexamethasone and two devices that release fluocinolone acetonide, Retisert (Bausch & Lomb, Rochester, NY), and Iluvien (Alimera Science, Alpharetta, GA). Each has different physical characteristics and duration effect and has been approved for different indications. Herein we provide a summary of the current clinical knowledge regarding these implants.

## 2. Dexamethasone Drug Delivery System (DDS)

Ozurdex (Allergan Inc., Irvine. Ca) is a biodegradable polymer composed of a polylactic acid-glycolic acid matrix that dissolves completely* in vivo* and is eventually converted to carbon dioxide and water [2]. The implant contains 700 *μ*g of dexamethasone which is released to the vitreous cavity over a six-month period. It is administered via a 22-gauge injecting applicator through the pars plana. It can be administered in an office setting. Animal studies have shown that the peak concentration is reached in the retina and vitreous at day 60 and is detectable for 6 months with minimal systemic absorption [5]. After the first two months, the steroid concentration declines until month 4, where it maintains a lower concentration until month 6. The pharmacokinetic profile is similar between vitrectomized and nonvitrectomized eyes [6]. This is important as other medications such as triamcinolone acetonide are less effective in vitrectomized eyes due to faster clearance from the ocular tissues [7]. The implant is contraindicated in patients with periocular infections and advanced glaucoma and in patients whose posterior lens capsule is not intact because of the risk of implant migration into the anterior chamber.

Ozurdex has been approved by the FDA for the treatment of macular edema secondary to branch or central retinal vein occlusion, as well as for the treatment of noninfectious posterior uveitis. Haller et al. [8] published a phase III trial of Ozurdex for macular edema secondary to branch or central retinal vein occlusion. The study included 1,267 patients and evaluated two concentrations of dexamethasone and a sham injection group. Patients receiving the dexamethasone implant had a statistically significant improvement in vision compared to the sham group. The greatest improvement was seen at day 60 with the 700 *μ*g implant, with 29% of patients achieving a 15-letter improvement in vision. The proportion of eyes achieving at least a 15-letter improvement from baseline was significantly greater in patients receiving the injection at months 1 and 3, but no difference was seen at 6 months [9]. Cataracts were not increased in any group, and although 30% of eyes were treated with intraocular pressure (IOP) lowering medication, the IOP returned to baseline after 6 months of the procedure in all groups. After 6 months, all study patients were eligible to receive an implant if they experienced a drop in vision or persistent macular edema [10]. The same efficacy and a similar effect on the IOP were seen in the 997 patients who received an implant after 6 months. The only difference was seen in cataract progression, which occurred in 29.8% of patients who received two implants versus 5.7% in patients who had previously belonged to the sham group. However, only one patient required cataract surgery.

A systematic review published by Pielen et al. [11] compared anti-VEGF agents (ranibizumab, bevacizumab, aflibercept) versus steroids (triamcinolone and Ozurdex) for macular edema in CRVO or BRVO. All anti-VEGF agents showed a better visual acuity gain compared to steroids at month 12. The downside was that anti-VEGF therapy requires more frequent injections (around 8 injections per year, compared to 2 injections in the steroid group). IOP increase and cataract progression were also significantly higher in the patients treated with steroids compared to patients treated with anti-VEGF agents. Prospective studies comparing ranibizumab versus Ozurdex are ongoing (COMO and COMRADE (http://www.clinicaltrials.gov)). The dexamethasone implant may be of value in vitrectomized eyes, where anti-VEGFs have shown significantly reduced half-life compared to nonvitrectomized eyes in previous reports [12], although some authors argue there is no difference [13].

The phase III trial for Ozurdex for the treatment of noninfectious intermediate or posterior uveitis was published by Lowder et al. in 2011 [14]. This study included 229 patients and compared two concentrations of dexamethasone versus sham. The proportion of patients with a vitreous haze score of 0 at 8 weeks was significantly higher with the 700 *μ*g implant, and this effect persisted through week 26. Best corrected visual acuity was also significantly better in the dexamethasone treated eyes compared to sham. No significant difference was seen in cataract progression or in the proportion of patients with an IOP > 25 mmHg between the groups. The duration of the study was 6 months and no repeat injections were performed. Ozurdex is approved by the FDA for the treatment of posterior segment noninfectious uveitis.

Ozurdex has also been proposed as treatment for diabetic macular edema (DME). A subgroup analysis of 171 patients with DME that received an Ozurdex implant showed that visual acuity gain was significantly higher compared to observation (33% gained 2 or more lines at 90 days versus 12% in the observation group). Some case series have been published stating that Ozurdex is useful in recalcitrant DME [15, 16]. However, results of Allergan-sponsored prospective studies with repeated injections (NCT00168389, NCT00168337) have not been published. Currently, a study is underway to use Ozurdex for DME in vitrectomized patients (NCT01788475). Ozurdex has been approved by the FDA for the treatment of adults with diabetic macular edema who are pseudophakic or who are scheduled for cataract surgery.

## 3. Fluocinolone Acetonide Implant

Retisert (Bausch & Lomb, Rochester, NY) contains a 0.59 mg pellet of fluocinolone acetonide (FA) in a nonbiodegradable polyvinyl acetate/silicone laminate. It is implanted through a pars plana sclerotomy and secured by a suture in the sclera. This procedure is performed in the operating room. It releases fluocinolone acetonide for up to 3 years [17]. Ocular drug levels are stable over a year with no evidence of systemic absorption. There is no data regarding pharmacokinetics in vitrectomized eyes, although drug levels were measured in rabbit eyes with a C3F8 gas bubble with no significant changes [18]. Similar levels of the drug are seen not only at the level of the RPE but also in the lens and iris-ciliary body. Retisert is approved by the FDA for the treatment of chronic noninfectious posterior uveitis. It is contraindicated in active viral, bacterial, mycobacterial, and fungal eye infection. It is the most expensive of the devices discussed in this paper (around USD $20,000 versus $2,000 for Ozurdex and $8,000 for Iluvien). A comparative cost effectiveness analysis is not yet available.

The pivotal trial evaluating Retisert in the Unites States randomized 278 patients with noninfectious posterior uveitis to an implant containing either 2.1 mg or 0.59 mg of FA [19]. After implantation, uveitis medications and systemic immunosuppression were tapered within a six-week period. The uveitis recurrence rate decreased from 51.4% (includes the two types of implants) to 6.1% in the first 34 weeks after implantation. Eyes that did not receive an implant had an increased rate of recurrences from 20.3% to 42%. Results from three-year followup showed recurrence rates of 4, 10, and 20% at 1, 2, and 3 years [20]. At two years, visual acuity was significantly better in the implanted eyes (that difference was lost at three years). However, 93% of implanted phakic eyes required cataract surgery (compared to 20% in nonimplanted eyes). 37% of eyes required glaucoma surgery and 75% required pressure lowering medications. It has been proposed that the implant loses its effect after about 2.5–3 years, when it can be exchanged for a new implant if inflammation recurs. In some cases the implant can become dissociated from its strut during the procedure [21, 22]. A newly designed implant has been released in March 2013 to decrease the risk of medication reservoir dissociation, but long-term data on whether this complication will occur is unavailable at this time.

A multicenter, prospective clinical trial was performed comparing Retisert to standard of care in posterior uveitis [23]. Implanted eyes had significantly fewer recurrences (18.2% in the implant group versus 63.5% in the standard of care group). Uveitis recurrences occurred significantly later during followup. The incidence of cataract and glaucoma was similar to previous reports and there were no nonocular complications. Patients in the standard of care group had a 26% incidence of systemic treatment-related adverse effects. Visual acuity decreased between months 15 and 18 in the implant group probably due to the high incidence of cataract but was similar between both groups at month 24, with no decrease from baseline.

The MUST trial was a large prospective trial (255 patients, 479 eyes with uveitis) to compare the efficacy of the FA implant versus systemic immunosuppression with a followup of 24 months [24]. Visual acuity improvement was comparable between both groups, with a gain of 6 letters in the implant group and of 3.2 letters in the systemic therapy group at 24 months (*P* = 0.16). Control of uveitis was more frequent in the implant group (88% versus 71%, *P* = 0.0001). Although the number of patients with macular edema significantly decreased in the implant group at 6 months, the proportion of patients with macular edema was similar between both groups at 24 months. The implant group had a much higher rate of cataract surgery (80% versus 31% in the systemic treatment group) and glaucoma surgery (26.2 versus 3.7% in the systemic treatment group). Systemic infection requiring prescription therapy was lower in the implant group (0.36 events/person-year in the implant group versus 0.60 in the systemic therapy group, *P* = 0.034), but the risk of hospitalization was similar between both groups. Health-related quality of life and health utility scores increased in both groups, slightly favoring implant therapy (not significant).

Retisert has been proposed as treatment for diabetic macular edema. The largest trial randomized 196 participants to the FA implant versus laser treatment [25]. The percentage of eyes that gained 3 or more lines of vision was significantly higher in the implant group versus laser treatment at 6 months (16.8% versus 1.4%, resp.) and two-year followup (31.8% for the implant versus 9.3% for the laser group). At three years the proportion of patients who gained three or more lines of vision was 31.1% in the implant group versus 20% in the laser group (not significant). The implant group had a significantly higher rate of ocular complications with 91% of phakic eyes requiring cataract surgery and 33.8% requiring glaucoma surgery at 4 years. Although the results were comparable to those obtained with ranibizumab (RISE and RIDE studies [26]) where 36.8% to 51.2% of patients gained three or more lines of vision, the safety profile seems to be better with ranibizumab regarding cataract (incidence of 0.8%) with no increase in IOP. Retisert is not FDA approved for diabetic macular edema.

Retisert was evaluated in a prospective case series of 23 patients with CRVO [27], but a significant increase in visual acuity compared to baseline was not seen at three-year followup, despite improvements in central retinal thickness and 50% of eyes gaining 10 or more letters of vision. Ocular adverse events were similar to previous studies.

## 4. Fluocinolone Acetonide Insert

Iluvien (Alimera Science, Alpharetta, GA) is a smaller, nonbiodegradable cylindrical tube with a central drug-polymer matrix that releases 0.19 mg of fluocinolone acetonide into the vitreous cavity. It is inserted intravitreally via a 25-gauge needle in the same manner as in intravitreal injection and can be done in the office setting. It releases small doses of fluocinolone acetonide for at least 3 years. No systemic absorption has been documented [28].

The efficacy of Iluvien was evaluated in the FAME (Fluocinolone Acetonide for Diabetic Macular Edema) A and B studies which were two randomized, double-masked, sham injection-controlled multinational trials [29, 30]. Patients (*n* = 956) were randomized to one of two fluocinolone acetonide (FA) insert concentrations (0.2 *μ*g/day or 0.5 *μ*g/day) or sham. Patients could receive rescue laser photocoagulation during the study if there was persistent macular edema. After one year they could receive a second implant if their vision decreased or foveal thickness increased. Clinicians were allowed to use other nonprotocol drugs (such as anti-VEGF therapy or intravitreal triamcinolone) if they felt patients were experiencing no improvement. These medications were recorded but patients were not exited from the study. The primary endpoint was a gain of 15 or more letters at 24 months. In each of the insert groups 28% of patients achieved this goal versus 16% in the sham group (*P* = 0.002). Mean change from baseline BCVA was also significantly higher in the insert groups compared to sham. There was a drop in visual acuity between months 9 and 18 in the insertion group due to the development of cataract which later improved with surgery.

Foveal thickness was significantly less in the insert groups compared to sham at all time points except at 36 months. Significantly fewer patients required rescue laser photocoagulation in the insert groups (40%) versus sham (60%), *P* < 0.001. Regarding other nonprotocol rescue treatments (anti-VEGF, intravitreal triamcinolone), they were administered significantly more in the sham group (28.6%) compared to the insert groups (12.5–13.9%). A subgroup analysis showed that the implant was significantly better compared to sham in diabetic macular edema of more than 3 years of onset (34% versus 13.4%), but all groups were similar in edema of <3 years duration. There was no difference in the effectiveness of both concentrations of the insert, so the one with the lowest drug concentration is commercially available.

Regarding adverse events at 36 months, phakic patients who received the 0.2 *μ*g/day implant developed cataracts in 81.7% versus 50.7% in the sham group and 80% required cataract surgery versus 27.3% in the sham group. Raised IOP was present in 37.1% in the insert group versus 11.9% in sham. Incisional IOP lowering surgery was performed in 4.8% of patients in the implant group (0.2 *μ*g/day) versus 0.5% in the sham group.

Iluvien is approved for use in several European countries (Austria, France, Germany, Portugal, and Spain and is pending approval in Italy) for the treatment of impairment of vision associated with chronic DME that is insufficiently responsive to available therapies [28]. It has yet to receive approval by the FDA for use in the United States. No data is available for the treatment of macular edema from CRVO or BRVO. Pivotal trials are underway to evaluate the effect of Iluvien in the treatment of noninfectious posterior uveitis, although no data is available at this moment.

## 5. Conclusion

Sustained-release corticosteroid treatment has shown to be a promising strategy for macular edema due to retinovascular disease (i.e., diabetes and retinal vein occlusion) and for the treatment of noninfectious posterior uveitis. Different types of implants are available, with various mechanisms, duration, and side effects. Ample experience has shown the effectiveness of Ozurdex, but in patients with chronic disease punctuated by recurrences, a treatment duration longer than 6 months is desirable. The fluocinolone acetonide implants have demonstrated increased duration of efficacy, but side effects such as cataract and glaucoma including the potential need for surgical treatment are considered. Further studies are needed to determine the indications for each of these implants, as well as long-term results, including the effects of receiving multiple sustained release steroids over time.
